# Machine learning model to predict obesity using gut metabolite and brain microstructure data

**DOI:** 10.1038/s41598-023-32713-2

**Published:** 2023-04-04

**Authors:** Vadim Osadchiy, Roshan Bal, Emeran A. Mayer, Rama Kunapuli, Tien Dong, Priten Vora, Danny Petrasek, Cathy Liu, Jean Stains, Arpana Gupta

**Affiliations:** 1 Vatche and Tamar Manoukian Division of Digestive Diseases, Los Angeles, USA; 2 UCLA Microbiome Center, Los Angeles, USA; 3 G. Oppenheimer Center for Neurobiology of Stress and Resilience, Los Angeles, USA; 4 Department of Urology, David Geffen School of Medicine, Los Angeles, USA; 5 Division of Gastroenterology, Hepatology and Parenteral Nutrition, VA Greater Los Angeles Healthcare System, Los Angeles, CA USA; 6 Department of Mathematics, California Institute of Technology, Pasadena, USA; 7 G. Oppenheimer Family Center for Neurobiology of Stress and Resilience, Vatche and Tamar Manoukian Division of Digestive Diseases, David Geffen School of Medicine at UCLA, CHS 42-210 MC737818, 10833 Le Conte Avenue, Los Angeles, CA USA

**Keywords:** Systems biology, Diseases

## Abstract

A growing body of preclinical and clinical literature suggests that brain-gut-microbiota interactions may contribute to obesity pathogenesis. In this study, we use a machine learning approach to leverage the enormous amount of microstructural neuroimaging and fecal metabolomic data to better understand key drivers of the obese compared to overweight phenotype. Our findings reveal that although gut-derived factors play a role in this distinction, it is primarily brain-directed changes that differentiate obese from overweight individuals. Of the key gut metabolites that emerged from our model, many are likely at least in part derived or influenced by the gut-microbiota, including some amino-acid derivatives. Remarkably, key regions outside of the central nervous system extended reward network emerged as important differentiators, suggesting a role for previously unexplored neural pathways in the pathogenesis of obesity.

## Introduction

The obesity epidemic has emerged as a major public health crisis nationally and internationally^1,2^. In addition to costing the healthcare system hundreds of billions of dollars, there are countless associated negative health outcomes including cancers, endocrinological disorders, musculoskeletal disorders, and a well-documented increase in premature mortality from cardiovascular disease^3^. Additionally, the distinction between overweight and obese is becoming increasingly important as many studies have demonstrated a dose-dependent relationship between excess weight or body mass index (BMI) and health outcomes^4,5^.

The pathophysiology of obesity remains complex, representing a derangement of energy homeostasis and gut endocrine signaling, especially within the context of aberrant insulin sensitivity and regulation, in addition to disruptions in the fine balance of pro- and anti-satiety signals in the gut^6,7^. In brief, gut hormones such as ghrelin produce hunger and cravings^8,9^, while hormones such as glucagon like peptide (GLP)-1^10^ and peptide tyrosine tyrosine (PYY)^11^ trigger satiety. External factors, such as the gut microbiota, can disrupt this carefully orchestrated homeostatic energy balance. For example, spore forming microbes found in the human gut microbiome can influence enteroendocrine cells of the gut to release more or less GLP-1 in response to microbiota-derived secondary bile acids^12^.

Obesity, however, is just as much a disorder of the endocrine system as it is of the brain, especially with respect to the extended reward network, which is responsible for processing rewarding stimuli and food-seeking behaviors. Key regions of the extended reward network that have been implicated include those related to salience, executive control, core reward, sensorimotor, and emotional regulation-related processes^13–17^. Despite the robust body of neuroscience research on obesity, investigations have almost exclusively focused on understanding how the obese brain differs from the non-obese brain; no studies have investigated central nervous system (CNS) changes that differentiate obese from overweight individuals. In addition to identifying anatomical and functional alterations in specific brain regions, more recent efforts have focused on identifying alterations in brain network properties^18^. To assess brain connectivity, graph theory has been leveraged to perform complex network analysis. In this way, we can quantify the anatomic and functional contributions to information flow within the context of a global, whole brain network^19–22^. Previous investigations used this approach to explore CNS alterations between high BMI and normal BMI brain connectivity^23,24^.

Perhaps attributed to the feasibility of more computationally rigorous approaches, combined with an overwhelming amount of data, the last decade has seen an explosion of research leveraging systems-based approaches to understanding complex human disease states, such as obesity. One such example has been to view obesity as a brain-gut disorder, with the gut microbiome also likely to modulate these interactions. Most of the high quality evidence to suggest a role for these brain-gut interactions have been from pre-clinical studies, or primarily cross-sectional clinical studies^25,26^. It is important to note that there has been limited progress in developing effective, long-lasting treatments for obesity^27,28^. This is likely driven by both the complex pathophysiology of obesity, combined with the failure to view obesity through a systems biology lens, incorporating both neuroimaging and gut metabolite data. Recent advances in machine learning have allowed for the ability to build robust, predictive models that can distill very large amounts of data into a smaller number of highly impactful features. Here, we use a machine learning approach to leverage the enormous amount of neuroimaging and fecal metabolomic data to better understand key drivers of the obese compared to overweight phenotype.

## Methods

### Study participants

The total sample was comprised of 117 right-handed healthy adult (age ≥ 18) volunteers (36 males and 81 females). A medical exam and clinical assessment that included a modified Mini-International Neuropsychiatric Interview Plus 5.0 (MINI) (27) was administered to confirm the absence of significant medical or psychiatric conditions. Subjects were excluded from participating in the study for any of the following reasons: pregnant or lactating, substance abuse, abdominal surgery, tobacco dependence (smoked half a pack of cigarettes or more daily), extreme strenuous exercise (> 8 h of continuous exercise per week), current or past psychiatric illness, and major medical or neurological conditions. Subjects taking medications that interfere with the central nervous system (full dose antidepressants including SSRI, NSRIs, sedatives or anxiolytics) or regular use of analgesic drugs (including narcotics, opioids, and α2-δ ligands) were excluded. Since female sex hormones such as estrogen are known to effect brain structure and function, in this study we used women who were premenopausal and who were scanned during the follicular phase of their menstrual cycles as determined by self-report of their last day of the menstrual cycle.

Subjects with hypertension, diabetes, or metabolic syndrome were also excluded from the study to minimize confounding effects from our findings. For the same reason, subjects with eating disorders such as anorexia or bulimia nervosa were also excluded. For the purpose of our analyses, we used BMI cutoffs to define our groups: Overweight individuals had a BMI ≥ 25 but < 30, obese individuals had a BMI > 30. Individuals with normal BMIs or who had BMIs that would be considered underweight were excluded from our analysis (BMI < 25). No subjects exceeded 400lbs due to MRI scanning weight limits.

All procedures complied with the principles of the Declaration of Helsinki and were approved by the Institutional Review Board at UCLA’s Office of Protection for Research Subjects (approval numbers 11-000069 and 12-001802). All subjects provided written informed consent.

### MRI acquisition

A 3.0 T Siemens Trio scanner was used to perform whole brain structural, and diffusion tensor (DTI) magnetic resonance imaging. Noise reducing headphones were used. Automated data processing and computational workflows for structural and diffusion tensor imaging data were designed and implemented in collaboration with the University of Southern California Laboratory of Neuroimaging (LONI) Pipeline (pipeline.loni.usc.edu).

#### Structural gray-matter

For registration purposes, a high resolution structural image was obtained from each subject using a magnetization-prepared rapid acquisition gradient-echo sequence, repetition time = 2200 ms, echo time = 3.26 ms, structural acquisition time = 5 m 12 s, slice thickness = 1 mm, 176 slices, 256*256 voxel matrix, 1 mm voxel size.

#### Anatomical connectivity (DTI)

Diffusion-weighted MRIs (DWIs) were acquired according to two comparable acquisition protocols. Specifically, DWIs were acquired in either 61 or 64 noncolinear directions with b = 1000 s/mm^2^, with 8 or 1 b = 0 s/mm^2^ images, respectively. Both protocols had a TR = 9400 ms, TE = 83 ms, and field of view (FOV) = 256 mm with an acquisition matrix of 128 × 128, and a slice thickness of 2 mm to produce 2 × 2 × 2 mm^3^ isotropic voxels.

### MRI preprocessing and quality control

#### Structural gray-matter

Structural T1-image segmentation and regional parcellation were conducted using FreeSurfer ^29,30^ following the nomenclature described in Destrieux et al. ^31^. This parcellation results in the labeling of 165 regions, 74 bilateral cortical structures, 7 subcortical structures, the midbrain, and the cerebellum^32^.

#### Anatomical connectivity (DTI)

Diffusion weighted images (DWI) were corrected for motion and used to compute diffusion tensors that were rotationally re-oriented at each voxel. The diffusion tensor images were realigned based on trilinear interpolation of log-transformed tensors as described in Chiang et al. ^33^ and resampled to an isotropic voxel resolution (2 × 2 × 2 mm^3^). White matter connectivity for each subject was estimated between the 165 brain regions using DTI fiber tractography ^32^, performed via the Fiber Assignment by Continuous Tracking (FACT) algorithm ^34^ using TrackVis (http://trackvis.org).

### Anatomical MRI network construction

#### Connection matrix

Regional parcellation and tractography results were combined to produce a weighted, unidirected connectivity matrix. The final estimate of white matter connectivity between each of the brain regions was determined based on the number of fiber tracts intersecting each region. Weights of the connections were then expressed as the absolute fiber count divided by the individual volumes of the two interconnected regions ^21^.

#### Computing network metrics

The Graph Theory GLM toolbox (GTG) (www.nitrc.org/projects/metalab_gtg) and in-house matlab scripts were applied to the subject-specific anatomical brain networks to compute three local weighted network metrics indexing centrality. The network metrics are described below ^22,35–37^.

Measures of centrality quantify the importance of a region’s influence on communication and information flow in large-scale brain networks. These measures include strength, betweenness centrality and eigenvector centrality. Strength represents the number of connections (fiber tracts) a given brain region has, factoring in the “weight” of each connection and reflects a brain region’s total level of impact in the network. Betweenness centrality describes degree to which a brain region lies on the shortest path between two other regions. Acting as way stations, regions with high betweenness centrality are topologically primed to control communication between other regions^38^. Eigenvector centrality reflects how connected a given brain region is to other brain regions with high centrality (greater number of fiber tracts) and is a measure of a region’s overall influence on the network^39^.

### Stool collection and processing

Fecal samples were aliquoted under liquid nitrogen and shipped to Metabolon for processing and analysis as a single batch on their global metabolomics and bioinformatics platform. Data was curated by mass spectroscopy using established protocols and software as previously described^40^. The samples of stool were all collected within a week of the subjects’ MRIs.

### Machine learning model

A balanced, binary classification label was defined using clinical information about each participant’s weight.

For both the metabolite and brain DTI network metric datasets, the number of variables (987 and 2156 respectively) greatly outnumber the sample size of 117. Machine learning models containing this many feature with a comparatively smaller sample size often contain uninformative variables that lower the model’s classification accuracy. In order to arrive at an optimal, reduced feature space that gave a superior classification, a feature selection technique known as Recursive Feature Elimination (RFE) was utilized with cross validation. Previous work has shown RFE using a Support Vector Machine Estimator (SVM-RFE) to be effective at small sample size and high dimensional data modeling^41^.

RFE is a wrapper feature selection technique that essentially performs backwards selection on the predictors. A linear model is initially trained on the entire set of features, and iteratively, the features with the lowest weight are removed until a certain target of features to be kept is met. We used a Support Vector Machine (SVM) with a linear kernel as our model for RFE.

In this study, two different types of cross validation were used. K-fold cross validation is a resampling procedure that can be used to evaluate a machine learning model’s performance on a limited dataset. In this process the dataset is partitioned into k different, equally sized, subsets, which are termed folds. Of these k folds, a single one is isolated as a test data set while the rest of the data are designated for training. This process is repeated k times, with a different fold designated as test data at each iteration. Here, we used ten-fold cross validation and leave-one-out (LOO) cross validation, which involves iteratively isolating a single sample as test data and using the rest as training data^42^.

To determine the best target number of features to keep using RFE, a candidate range from 1 feature, all the way to the entire feature space was considered. RFE was performed with LOO cross validation for each candidate. The number of features that recorded the highest average test data accuracy was determined to be the optimal number of features to keep.

Using this optimal number, RFE was then performed again with ten-fold cross validation To determine the final feature subset, a voting strategy was used^43^. For each fold, a different subset of features was selected by RFE. Each time a feature was included in a fold’s RFE selection, it received a count, or “vote.” If “n” were the optimal number of features to keep, then the final subset of selected features was determined to be the top “n” features by votes across folds^43^.

This process was performed on both the metabolite and brain DTI datasets to arrive at minimal feature sets that maximized accuracy.

To further assess the ability of these chosen variables to classify obesity, SVM with a linear kernel, ridge classifier, and logistic regression models were created and trained on just these features. In addition to the isolated variables, patient age and sex were also included as model predictors. For the metabolite model, patient diet was included as well. Logistic regression and a ridge classifier, being different models than the one used as an estimator for RFE, were used to ensure robustness and linear separability of the data. The predictive ability of each of these final models was assessed using LOO cross validation.

Finally, a combined model was created by merging the top 90 percent of features by absolute value of model weight, from each of the final brain and metabolite models. This model was also assessed using leave one out cross validation.

For each model, in addition to overall accuracy, precision and recall were calculated as well. Precision is defined as the number of true positives over the number of true positives plus the number of false positives. Recall is defined as the number of true positives over the number of true positives plus the number of false negatives. In other words, precision is the proportion of predicted trues that were actually true, and recall is the proportion of actual trues that were correctly predicted as true by the model. Precision and recall were calculated for both the very obese and obese classes.

As an additional way to ensure that overfitting was not occurring, a permutation test was performed by randomly shuffling the labels and retraining all three (brain, metabolite, and combined) models^44^. With the null hypothesis that there is no difference between the original model’s test accuracy versus the test accuracy after shuffling the labels, this test illustrates whether the model actually learned based on the particular relationship between the features and the label, or otherwise simply fit very closely to our high dimensional feature data with a small sample size. If the latter occurred, then we would see that the model would still have an above average test accuracy or have overfit and learned something from this random data.

**Figure 1:**
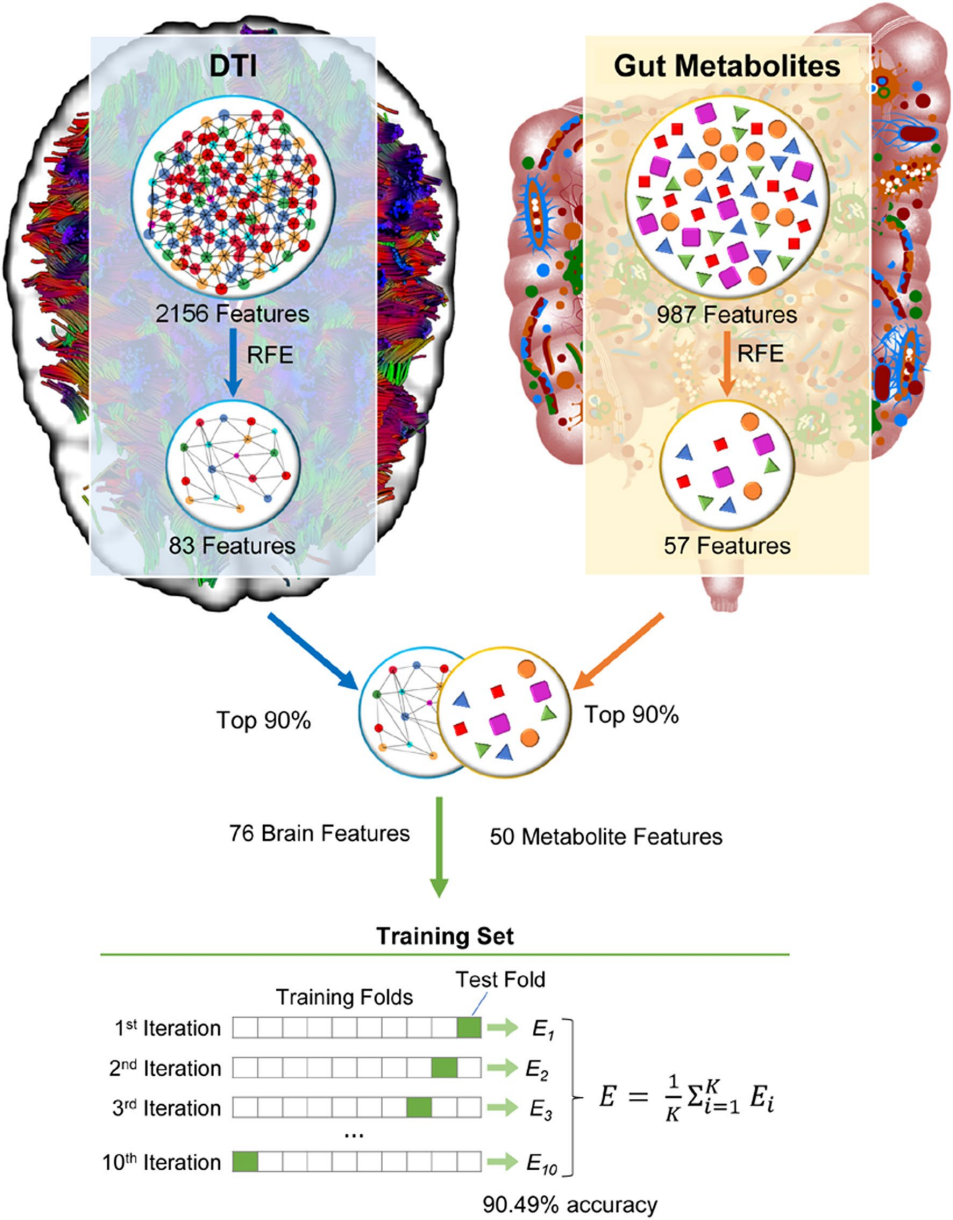
Study overview. In this study, we distil over three thousand unique brain microstructural and gut metabolite data into 76 brain and 50 metabolite features. These features were then used to create a machine learning model that could predict if patients were overweight or obese with over 90% accuracy.

Figure 1 provides a high-level overview of the methodology.

## Results

### Sample characteristics

The total sample (N = 117) included 64 obese individuals (females = 47, males = 17), mean age = 33.203125, standard deviation 10.2457, and 53 overweight individuals (females = 34, males = 19) mean age = 31.4528, standard deviation = 10.964. Clinical characteristics are summarized in Table 1.

**Table 1:** Clinical characteristics.

Column name	Overweight (n = 53)	Std	Obese (n = 64)	Std	P-value	T-value
Diets	American: 12 Other: 41	–	American: 29 Other: 35	–	0.01024	− 2.6106
Sexes	Male: 19 Female: 34	–	Male: 17 Female: 47	–	0.28261	1.07951
Ages	28.0	10.964	31.0	10.246	0.37888	0.88335
BMI	27.60274	1.61979	34.52796	3.79251	0.0	12.2843

### Recursive feature elimination results

**Figure 2:**
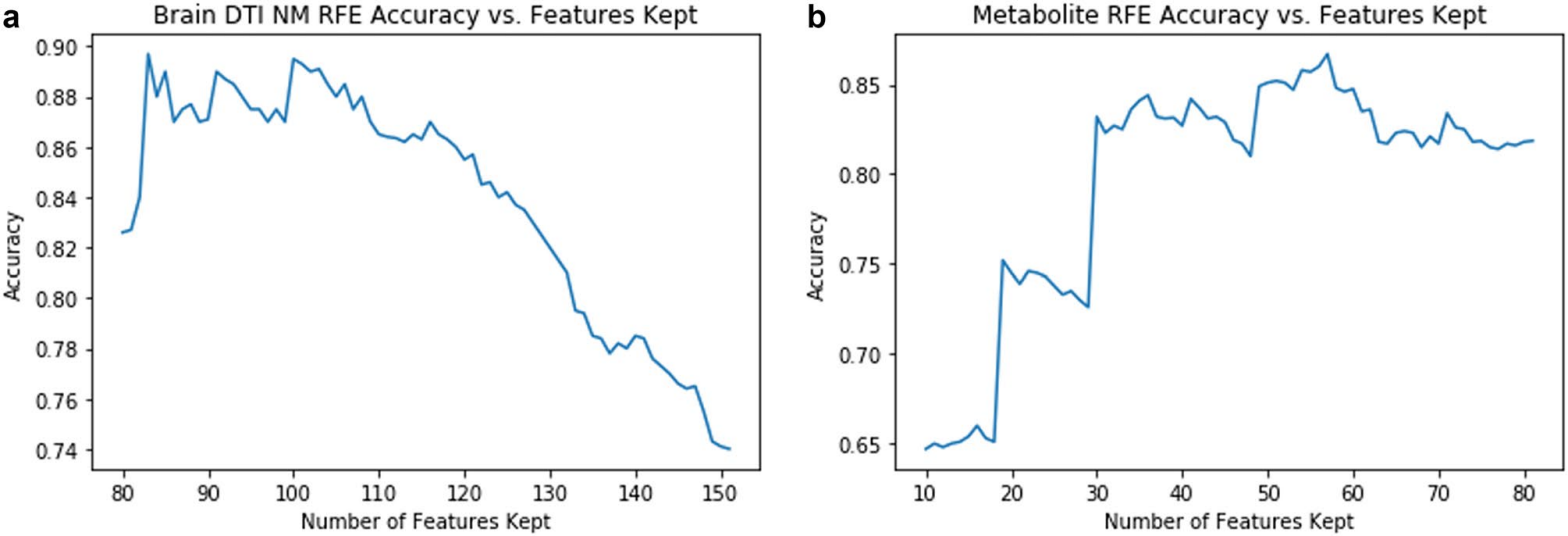
Recursive feature elimination graph of brain network metrics (**A**) and metabolites (**B**). A linear model is first developed incorporating all features and iteratively, the features with the lowest weight are removed. These graphs demonstrate the relationship between model accuracy and the number of features kept.

Our feature selection method identified 83 DTI features out of 2156 total variables for the brain model (Fig. 2A, SuppTable 1).

Out of 987 total metabolites, our feature selection method identified 57 variables for the metabolite model (Fig. 2B, SuppTable 2).

### Brain classifier

**Figure 3:**
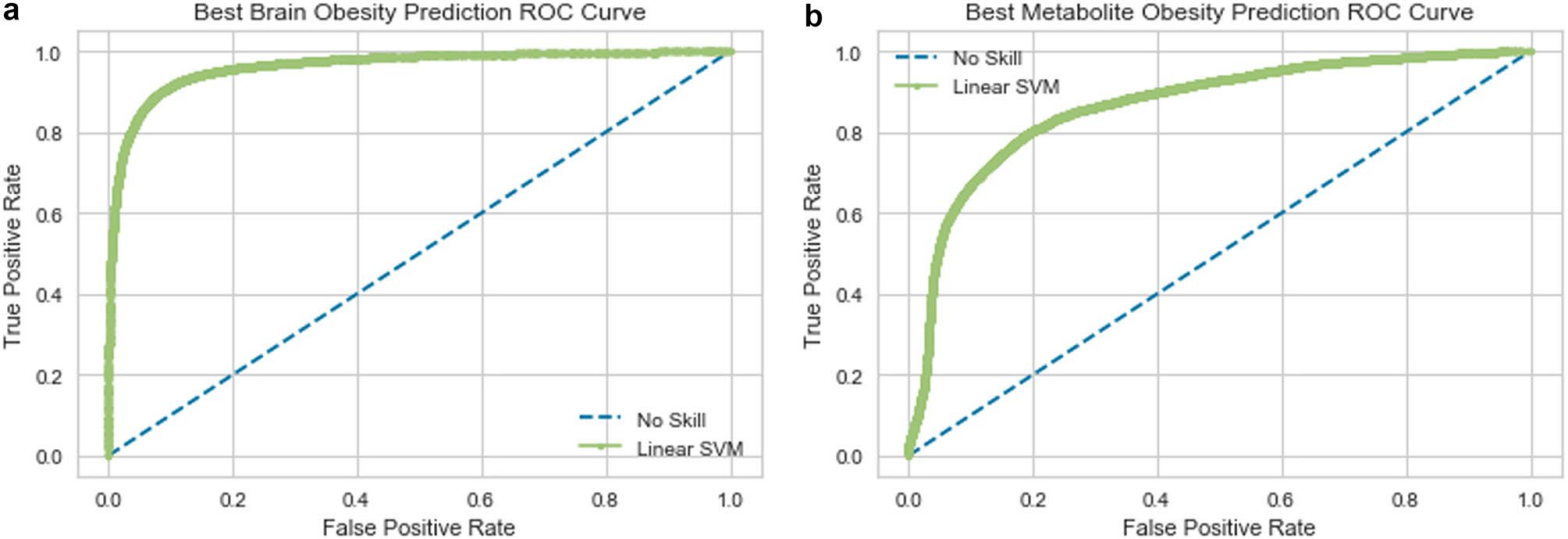
Receiver operating characteristic curve of support-vector machine model using brain features (**A**) exclusively, metabolite features exclusively (**B**).

An SVM model trained on this subset of DTI features identified by our RFE method achieved 90.25 percent accuracy in discriminating very obese patients from obese patients (Fig. 3A). The obese class (0 class) had a precision of 0.90 and a recall of 0.88. The extremely obese (1 class) had a precision of 0.91 and a recall of 0.92.

Interestingly, the 83 isolated DTI network metric features were all either node betweenness centrality or average path length measures of various parts of the brain. Out of the 83 isolated features, all of the corresponding brain regions were unique except for nine. The left triangular part of the frontal gyrus (L_InfFGTrip), left fronto marginal gyrus (of Wernicke) and sulcus (L_FMarG_S), right occipital pole (R_OcPo), right intraparietal (interparietal sulcus) and transverse parietal sulci (R_IntPS_TrPS), right transverse frontopolar gyri and sulci (R_TrFPoG_S), left inferior segment of the circular sulcus of the insula (L_InfCirIns), right inferior temporal sulcus (R_InfTS), Transverse frontopolar gyri and sulci(L_TrFPoG_S), and left caudate nucleus (L_CaN) each had both its node betweenness centrality and average path length measures in the identified feature set.

The three features with the largest positive weight, influencing the model most towards classification as extremely obese are average path length of the left pericallosal sulcus (AvPathLength__L_PerCaS), average path length of inferior occipital gyrus and sulcus (AvPathLength__L_InfOcG_S), and node betweeness centrality of the left temporal pole (NodeBWCent__L_Tpo). On the other hand, the three features with the largest negative weight, influencing our model towards classification as just obese are average path length of the right long insular gyrus and central insular sulcus (AvPathLength__RLoInG_CInS), average path length of the left middle-posterior part of the cingulate gyrus and sulcus (AvPathLength__L_MPosCgG_S), and posterior dorsal part of the cingulate gyrus (AvPathLength__PosDCgG) (SuppFigure 1).

### Metabolite classifier

An SVM model trained on this subset of metabolite features identified by our RFE method achieved 79.84 percent accuracy in discriminating very obese patients from obese patients (Fig. 3B). The obese (0 class) had a precision of 0.79 and a recall of 0.76. The extremely obese (1 class) had a precision of 0.81 and a recall of 0.83.

The three features with the largest positive weight, influencing the model towards classification as extremely obese were acesulfame, N-acetylisoleucine, and 1,2-dilinoleoyl-GPC (18:2/18:2). On the other hand, the three features with the largest negative weight, influencing the model towards classification as obese, were 1-oleoyl-GPC (18:1), pregnen-diol sulfate, and glyocholate (SuppFigure 2).

### Combined classifier

**Figure 4:**
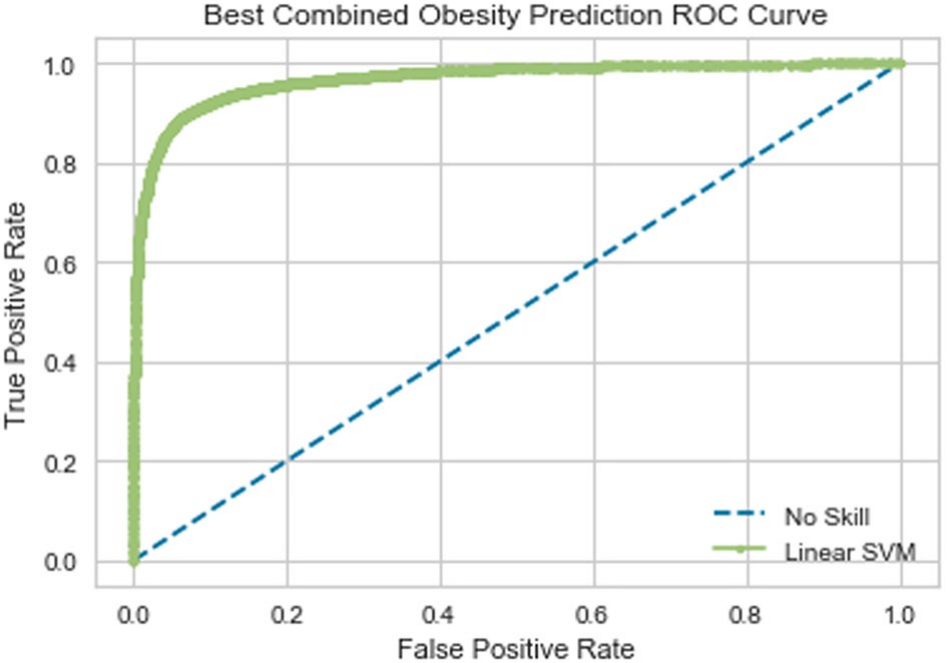
Receiver operating characteristic curve of support-vector machine model using combined brain and metabolite features.

Combining the top 90 percent of features by absolute value of weight from the two described models above yielded a new feature subset of 126 variables (50 metabolite variables and 76 brain dti nm variables) (Table 2). The SVM model trained on this subset of features slightly outperformed the brain classifier. It achieved 90.49 percent accuracy in discriminating very obese patients from obese patients (Fig. 4). The obese class (0 class) had a precision of 0.90 and a recall of 0.89, and the extremely obese class (1 class) had a precision of 0.91 and a recall of 0.92.

**Table 2:** Brain and metabolites features included in combined support-vector machine model of obesity.

Column name	Info	SVM weight	P-value	T-value
1-oleoyl-GPC (18:1)	Metabolite	− 3.20823e−05	0.3254347	− 0.9875739
Glycocholate	Metabolite	− 8.2497e−06	0.2450839	− 1.1683452
Pregnen-diol disulfate*	Metabolite	− 5.24364e−05	0.0766891	− 1.7862797
Nicotinate ribonucleoside	Metabolite	− 1.16909e−05	0.5345713	− 0.6229187
2-hydroxypalmitate	Metabolite	− 8.8495e-06	0.0279664	− 2.2259563
12-ketolithocholate	Metabolite	− 6.062e-07	0.6234562	− 0.4922853
11beta-hydroxyandrosterone sulfate (2)	Metabolite	− 6.3292e-06	0.1132935	− 1.5957305
*N*-stearoyl-sphinganine (d18:0/18:0)*	Metabolite	2.2885e−06	0.9127752	0.10978
Pantoate	Metabolite	− 5.9953e−06	0.0844496	− 1.7404801
5-hydroxyhexanoate	Metabolite	− 1.05904e−05	0.3068464	− 1.026423
Imidazole propionate	Metabolite	2.7469e−06	0.9937212	− 0.0078865
Agmatine	Metabolite	− 2.20964e−05	0.1105004	−1.6083507
*N*-methylproline	Metabolite	− 1.37931e−05	0.3443564	– 0.9495022
Arabitol/xylitol	Metabolite	− 2.07102e−05	0.1675273	− 1.3889653
Deoxymugineic acid	Metabolite	− 9.3404e−06	0.2403965	− 1.1800981
Theobromine	Metabolite	− 4.6408e−06	0.8264974	− 0.219696
Stachydrine	Metabolite	2.3491e−06	0.9693121	0.0385549
Docosahexaenoylcarnitine (C22:6)*	Metabolite	− 7.433e−06	0.0613478	− 1.8894642
Carboxyibuprofen	Metabolite	− 2.25928e−05	0.4496436	− 0.7586004
Hyocholate	Metabolite	− 2.7928e−06	0.4151871	− 0.817757
3,7-dimethylurate	Metabolite	− 4.1805e−06	0.7599397	− 0.3062874
7-methylxanthine	Metabolite	− 2.2189e−06	0.5315353	− 0.6275615
(R)-salsolinol	Metabolite	− 2.21099e−05	0.2229156	− 1.225434
5alpha-androstan-3alpha,17beta-diol disulfate	Metabolite	− 7.7926e−06	0.2266135	− 1.2156354
Curcumin	Metabolite	− 7.1795e−06	0.1972666	− 1.2968855
Docosadienoate (22:2n6)	Metabolite	− 6.8086e−06	0.2522873	− 1.1505912
Genistein	Metabolite	4.88011e−05	0.6114582	0.5093853
2′-deoxyadenosine 5′-monophosphate	Metabolite	1.2038e−06	0.785891	− 0.2722852
2-(4-hydroxyphenyl)propionate	Metabolite	1.64756e−05	0.2969804	1.0476857
Piperine	Metabolite	3.29415e−05	0.5037286	0.6707439
5-(2-Hydroxyethyl)-4-methylthiazole	Metabolite	− 9.7784e−06	0.9918073	0.0102906
Tyrosol	Metabolite	1.02517e−05	0.0755582	1.7932698
*N*-butyryl-leucine	Metabolite	5.3575e−06	0.0931012	1.6933227
I-urobilinogen	Metabolite	7.58174e−05	0.0195845	2.36738
*N*-(2-furoyl)glycine	Metabolite	2.14319e−05	0.3071521	1.0257716
OAHSA (18:1/OH-18:0)	Metabolite	− 8.6601e−06	0.7071483	0.3766223
AMP	Metabolite	− 2.3066e−06	0.2627772	− 1.1253659
Maltose	Metabolite	2.7173e−06	0.3679204	− 0.9039334
Sulfate of piperine metabolite C16H19NO3 (2)*	Metabolite	2.01842e−05	0.1394363	1.488196
gamma-Glutamylalanine	Metabolite	3.4847e−06	0.1697141	1.3817916
Docosahexaenoate (DHA; 22:6n3)	Metabolite	1.79368e−05	0.5487737	0.6013752
1-linolenoylglycerol (18:3)	Metabolite	− 2.6022e−06	0.5024215	0.6728043
1-oleoylglycerol (18:1)	Metabolite	5.74495e−05	0.2049111	1.2749096
Stigmastadienone	Metabolite	4.6773e−06	0.4839874	0.7021772
Linoleoyl-linoleoyl-glycerol (18:2/18:2) [1]*	Metabolite	5.4485e−06	0.0342553	2.1425781
Levulinate (4-oxovalerate)	Metabolite	2.1728e−06	0.2314901	1.2028887
Sucralose	Metabolite	8.6687e−06	0.116412	1.5819302
1,2-dilinoleoyl-GPC (18:2/18:2)	Metabolite	1.16417e−05	0.8407669	0.2013676
*N*-acetylisoleucine	Metabolite	5.4118e−06	0.02041	2.3512972
Acesulfame	Metabolite	2.93443e−05	0.222516	1.2264998
AvPathLength__R_LoInG_CInS	Somatosensory	− 0.0009482483	0.170343	− 1.3797417
AvPathLength__L_MPosCgG_S	Emotional Regulation	− 0.0009154738	0.0139718	− 2.4961668
AvPathLength__L_PosDCgG	Default Mode Network	− 0.0007531148	0.1134375	− 1.5950865
AvPathLength__R_InfFGOrp	Emotional Regulation	− 0.0005549889	0.4141841	− 0.8195216
NodeBWCent__R_PosCS	Somatosensory	− 0.0006092343	0.0692979	− 1.8336098
AvPathLength__L_InfCirIns	Somatosensory	− 0.0006176458	0.0244062	− 2.2807316
NodeBWCent__R_PosCG	Somatosensory	− 0.0005719311	0.6046272	− 0.5191882
NodeBWCent__L_RG	Emotional Regulation	− 0.0006018847	0.6067252	− 0.5161723
AvPathLength__R_PrCun	Default Mode Network	− 0.0005846671	0.0411581	− 2.0651356
NodeBWCent__L_ACgG_S	Emotional Regulation	− 0.0005199462	0.8344359	− 0.2094906
NodeBWCent__R_TrFPoG_S	Default Mode Network	− 0.000550803	0.4761666	− 0.7148237
AvPathLength__L_OcPo	Occipital	− 0.0005372031	0.3719004	− 0.8964214
AvPathLength__R_PosTrCoS	Interlobe	− 0.0005350337	0.1452779	− 1.4663656
NodeBWCent__R_AngG	Default Mode Network	− 0.0004671307	0.4646458	− 0.7336653
AvPathLength__R_PerCaS	Emotional Regulation	− 0.0004534601	0.0093628	− 2.6430109
AvPathLength__R_AOcS	Occipital	− 0.0004988374	0.0586744	− 1.9096126
NodeBWCent__L_CS	Somatosensory	− 0.0004482049	0.6791227	− 0.4147156
AvPathLength__R_SbOrS	Emotional Regulation	− 0.0005218257	0.0774365	− 1.7817068
AvPathLength__R_PaHipG	Emotional Regulation	− 0.0005000987	0.7399951	− 0.3326619
NodeBWCent__L_MOcG	Occipital	− 0.0004343127	0.1116088	− 1.6033124
NodeBWCent__R_MPosCgG_S	Emotional Regulation	− 0.000483703	0.3756011	− 0.8894819
NodeBWCent__L_PaHipG	Emotional Regulation	− 0.0004352314	0.5103618	− 0.6603312
AvPathLength__L_InfFGTrip	Emotional Regulation	− 0.000433419	0.2284636	− 1.2107763
AvPathLength__L_MACgG_S	Emotional Regulation	− 0.0003845632	0.0829239	− 1.7492025
NodeBWCent__L_PosCG	Somatosensory	− 0.0004257865	0.1377684	− 1.4945595
NodeBWCent__R_Hip	Emotional Regulation	− 0.0004448703	0.7521676	0.3165386
NodeBWCent__L_TrFPoG_S	Default Mode Network	− 0.0004187044	0.5716848	− 0.5671986
NodeBWCent__R_OcPo	Occipital	− 0.0003524324	0.9782904	− 0.0272716
AvPathLength__L_FMarG_S	Executive	− 0.0004036164	0.2131851	− 1.251794
AvPathLength__R_SupPrCs	Somatosensory	− 0.0002847931	0.3299016	− 0.9784583
NodeBWCent__R_Tha	Somatosensory	− 0.0003020804	0.4789275	− 0.7103462
NodeBWCent__R_MOcG	Occipital	− 0.0003067178	0.3078318	− 1.0243248
NodeBWCent__L_SupFG	Somatosensory	− 0.0002481055	0.5374476	0.6185325
NodeBWCent__R_SupFS	Somatosensory	− 0.0002719212	0.6366603	− 0.4736319
NodeBWCent__L_CaN	Core Reward	− 0.0002289915	0.0463734	− 2.0137213
NodeBWCent__L_PaCL_S	Somatosensory	− 0.0001960458	0.5392911	− 0.6157276
AvPathLength__L_InfFGOrp	Emotional Regulation	− 0.0002701086	0.69713	0.3901735
NodeBWCent__R_SbPS	Executive	− 0.0002149309	0.7591009	− 0.3073921
NodeBWCent__L_Hip	Emotional Regulation	3.375e−06	0.4010689	− 0.8428374
NodeBWCent__R_InfTS	Temporal	0.0001339698	0.5679843	0.5726727
NodeBWCent__L_Pu	Somatosensory	0.0001385234	0.5944141	0.5339394
NodeBWCent__L_HG	Temporal	0.0001799849	0.2125041	1.2536713
NodeBWCent__R_IntPS_TrPS	Executive Control	0.0002065054	0.912869	− 0.1096615
AvPathLength__R_TrFPoG_S	Default Mode Network	0.0001700514	0.6230364	− 0.4928811
NodeBWCent__R_InfFGTrip	Emotional Regulation	0.0001712679	0.4691462	0.7262744
AvPathLength__R_ShoInG	Salience	0.0002558495	0.1916472	1.313446
NodeBWCent__L_InfCirIns	Somatosensory	0.000298333	0.3661217	0.9073451
NodeBWCent__L_InfFGTrip	Emotional Regulation	0.0002452258	0.2790149	1.0876706
NodeBWCent__R_SupFG	Somatosensory	0.0003080609	0.2651615	1.1197311
AvPathLength__R_InfFGOpp	Emotional Regulation	0.0002563451	0.6090979	0.512767
NodeBWCent__L_SupFS	Somatosensory	0.0003189446	0.1007301	1.6546121
AvPathLength__R_OcPo	Occipital	0.0003393655	0.5872989	− 0.5442853
AvPathLength__R_Cun	Occipital	0.00039095	0.9285999	− 0.0898031
AvPathLength__R_ACirIns	Salience	0.0003916695	0.0878444	1.7215257
AvPathLength__L_SupTS	Temporal	0.0003220023	0.8840551	0.1461548
AvPathLength__R_TrTs	Temporal	0.0003712902	0.2822812	1.0802726
AvPathLength__L_SupPrCs	Somatosensory	0.0003569259	0.8959603	0.1310557
AvPathLength__L_InfPrCS	Somatosensory	0.000369516	0.6345674	0.4765773
AvPathLength__R_PosVCgG	Default Mode Network	0.0003904446	0.4358497	0.7819521
AvPathLength__L_TrFPoG_S	Default Mode Network	0.0003353048	0.8599587	− 0.1768227
AvPathLength__L_JS	Parietal	0.0004036897	0.5434692	0.6093882
NodeBWCent__L_MTG	Default Mode Network	0.0004397223	0.4000983	0.8445812
AvPathLength__L_InfTS	Temporal	0.000505792	0.4668332	0.730068
NodeBWCent__L_PRCG	Somatosensory	0.0004845014	0.0058443	2.8088183
NodeBWCent__L_FMarG_S	Executive	0.0005082393	0.2996283	1.0419329
NodeBWCent__R_SupCirInS	Salience	0.0005409756	0.2098923	1.2609129
AvPathLength__L_ACirIns	Salience	0.0005799921	0.253704	1.1471418
NodeBWCent__R_SupTGLp	Temporal	0.0005360867	0.0692834	1.8337069
NodeBWCent__R_CS	Somatosensory	0.0005749146	0.732848	0.3421693
NodeBWCent__L_SupTGLp	Temporal	0.0005546731	0.6895121	0.4005262
NodeBWCent__L_PrCun	Default Mode Network	0.0005835341	0.8516433	0.1874434
NodeBWCent__L_SupOcG	Occipital	0.000645764	0.1567469	1.4254113
AvPathLength__R_InfPrCS	Somatosensory	0.0006194332	0.4971359	0.6811658
NodeBWCent__L_Tpo	Temporal	0.0006079824	0.2851643	1.0737911
AvPathLength__L_InfOcG_S	Occipital	0.0006837278	0.049996	1.9808431
AvPathLength__L_PerCaS	Emotional Regulation	0.0009624533	0.5916009	0.538023

In this model, the features from the brain DTI dataset had significantly greater weights than those from the metabolite dataset. The three features with the largest positive weight, influencing the model towards classification as extremely obese were average path length of the left pericallosal sulcus (AvPathLength__L_PerCaS), average path length of inferior occipital gyrus and sulcus (AvPathLength__L_InfOcG_S), and node betweeness centrality of the Superior occipital gyrus (SupOcG).

**Figure 5:**
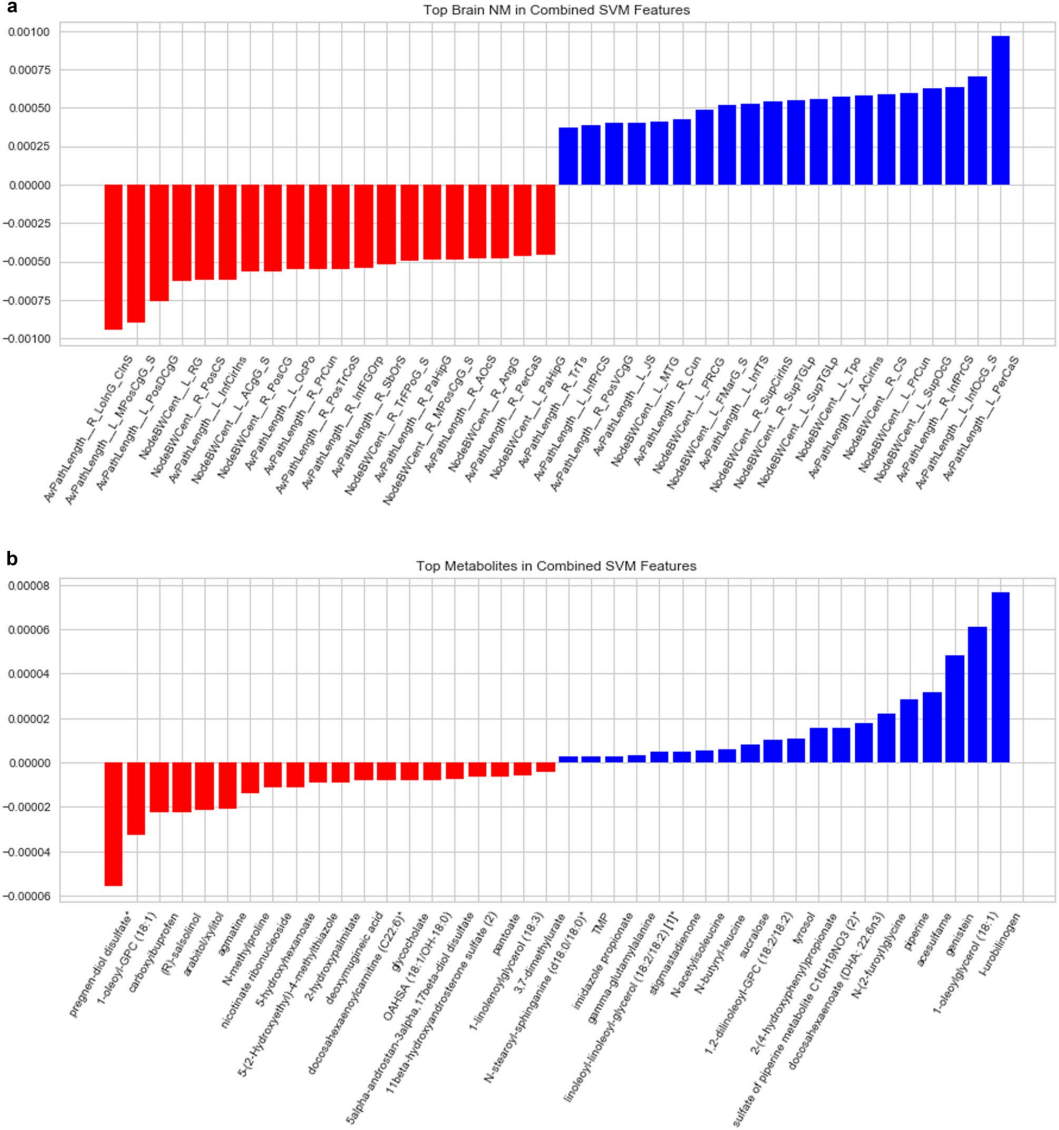
Top brain (**A**) and metabolite (**B**) features in combined support-vector machine model of obesity. The x-axis represents the brain and metabolite features of interest that contributed the most to the machine learning model—the magnitude of contribution is outlined on the y-axis, with a negative weight associated with the obese phenotype and a positive weight with the overweight phenotype.

The three features with the largest negatives weights were average path length of the right long insular gyrus and central insular sulcus (AvPathLength__RLoInG_CInS), average path length of the left middle-posterior part of the cingulate gyrus and sulcus (AvPathLength__L_MPosCgG_S), and posterior dorsal part of the cingulate gyrus (AvPathLength__PosDCgG) (Fig. 5A).

The most negative metabolite features in the combined model were pregen-diol sulfate, 1-oleoyl-GPC (18:1), and R-salsolinol. Lastly, the most positive metabolite features in this model were I-urobilinogen, 1-oleoylglycerol (18:1), and genistein (Fig. 5B).

### Permutation testing

For all three models, this null hypothesis was rejected. By shuffling the labels, the test accuracy with ten-fold cross validation decreased to worse than random (46 percent). This supports our claim that the models learned meaningful relationships between the features (metabolites and brain DTI) and the label (obesity level).

## Discussion

In this study, we demonstrate a machine learning approach that can successfully differentiate overweight from obese individuals using fecal metabolites and neuroimaging data, independently; however, combination of these two data parameters yields a more accurate classifier. Our results reveal a role for metabolites and brain regions of interest that have been previously investigated within the context of obesity, in addition to suggesting a role for previously unexplored metabolites and regions within this context. While not providing information about causality, our findings will be important for future, mechanistic studies, as this represents the first investigation of brain-gut interactions in differentiating obese from overweight individuals.

A substantial number of brain regions in the extended reward network, primarily the emotional regulation and somatosensory networks, emerged as important in differentiating obese from overweight individuals. These brain regions, most notably the inferior frontal gyrus, cingulate gyrus, and straight gyrus (emotional regulation), in addition to the postcentral gyrus, posterior insula, and paracentral lobule (somatosensory) have been previously described in multivariate analysis pattern classifiers that are able to differentiate normal weight from overweight individuals with a high degree of accuracy^23^. Only one region of the core reward network, the caudate nucleus, emerged as an important, albeit relatively minor, differentiator in our model. Although disruptions in core reward connectivity have been shown to be particularly important in differentiating normal from overweight phenotypes^23^, our results suggest that these disruptions are likely not the most important factors in differentiating overweight and obese individuals. Unexpectedly, regions outside of the extended reward network, primarily the default mode network, also emerged as important in differentiating obese from overweight individuals. Activity in the default mode network reflects baseline brain function, in addition to spontaneous self-reflection^45^, attention to internal stimuli^45^, and accounts for up to 80% of the brain’s energy use^46^. Research on the default mode network within the context of obesity is limited; though, our findings are consistent with a preclinical mouse model that demonstrates increased default mode network activity in overweight but not lean mice^47^. Taken together, our results suggest that obesity should not necessarily be interpreted as a more extreme version of the overweight phenotype, but rather as perhaps a different entity with a unique neuroimaging signature that is in many ways distinct from the overweight one.

Several amino acid derivatives (*N*-methylproline [from proline], imidazole propionate [histidine], agmatine [arginine], *N*-acetylisoleucine [isoleucine] and *N*-butyryl-leucine [leucine]) emerged as important in differentiating obese from overweight individuals. Previous work has linked amino acid and branched-chain amino acids in particular to obesity and related insulin resistance in both human and preclinical models, though these studies have been primarily performed using serum rather than fecal samples^48–51^. It is likely that the microbiome plays an important role in mediating this relationship; one study showed that mice transplanted with the microbiomes of human twin pairs discordant for obesity demonstrated differences in body composition, with the microbial communities in the obese mice showing increased metabolism of branched-chain amino acids^52^. Furthermore, a subset of the metabolites that emerged as some of the strongest differentiators between obese and overweight individuals in our data set are produced exclusively by gut microbes, including imidazole propionate^53^. L-urobilinogen, a metabolite produced in the intestine by bacterial reduction of bilirubin was also an important differentiator^54^. Previous studies have suggested a role for hyperbilirubinemia and obesity with respect to blood levels^54^.

With activity on serotonergic and glutaminergic neurotransmitters, agmatine is one of the handful of metabolites highlighted in our results that has been extensively studied with respect to its role as a neuromodulator^55^. Agmatine is also released from endogenous neurons in the peripheral nervous system and astrocytes in the central nervous system as a compensatory, protective mechanism in response to stress and inflammation^56^. Gut agmatine may interact with vagal afferents and contribute to the unique brain-gut signature of the obese and overweight phenotype. As many of the other significant metabolites have not been studies within the context of brain-gut interactions, we cannot conclude if these compounds communicate with the central nervous system in some way.

Our study focused on structural connectivity of brain regions and fecal metabolites. Future work may benefit from incorporating functional connectivity of brain regions or perhaps pairwise connectivity in characterizing key differences between overweight and obese individuals. Although we discussed the metabolites of interest within the context of the gut microbiome, we did not explicitly examine gut microbiota signatures (16S rRNA gene sequencing data) in this study. Future, more complex, machine learning models may benefit from the addition of different data sets to deepen our systems-based understanding of obesity and how it may be different from the overweight phenotype. We are unable to define the directionality and causality between fecal metabolites and alteration in brain connectivity with respect to obesity from this study; however, previous work has suggested bidirectional models for brain-gut-microbiome communication in obesity^57^. Machine learning tends to focus on the variability of features in order to explain the various outcomes. As a result, machine learning can eliminate features with low variance. This may not be correct in all situations as sometimes small variations can potentially drive large changes in the systems. To some extent, this bias can be eliminated by ensuring that the models being built and used are validated by subject matter experts. Although many of the fecal metabolites included in this model were not *on their own* able to differentiate between the overweight and obese phenotypes, the unique metabolome signature *was* able to significantly differentiate between the two groups, which underscores the impact of a machine learning approach to our systems understanding of obesity, which would otherwise not be captured in more traditional, strictly correlational analyses. Despite their predictive accuracy in classifying the data, machine learning algorithms cannot always entirely explain the true underlying processes, while also arriving at a final predictive model with the best set of features for optimizing accuracy. In this study, we differentiated our two groups by BMI; although BMI, which expresses the relationship between height and weight is the most widely used measure of obesity. Future studies may consider other measures of obesity such as waist–hip ratio or visceral adiposity in order to validate our current study.

To our knowledge, this is the first study to integrate microstructural neuroimaging and fecal metabolite data using a machine learning model to understand key differentiators and potential drivers of obese and overweight individuals. A growing body of evidence suggests that brain-gut interactions are important in driving the transition from being normal weight to overweight. Our results suggest that brain-directed signaling may be *more* important in obesity pathophysiology compared to overweight pathophysiology, as our machine learning model weighed alterations in brain connectivity as substantially more significant compared to fecal metabolites. This exploratory analysis supports previous preclinical and clinical investigations, while also revealing novel insights, which will be essential in driving discovery into previously unexplored avenues of brain-gut-microbiome interactions in obesity.

## Supplementary Information

Supplementary Table 1.

Supplementary Table 2.

## Data Availability

The datasets generated and/or analyzed during the current study are not publicly available due to the fact that the data collected are a part of an ongoing study but are available from the corresponding author on reasonable request.
