# Supplementary material for: Machine learning model to predict obesity using gut metabolite and brain microstructure data
Source: Sci Rep. 2023 Apr 4;13:5488. doi: 10.1038/s41598-023-32713-2 (PMC10073225; doi:10.1038/s41598-023-32713-2)
Supplement: Supplementary file 1 — Supplementary Table 1. [file 41598_2023_32713_MOESM1_ESM.docx]

SuppTable1

| Column Name | SVM Weight | P-Value | T-Value |
| --- | --- | --- | --- |
| AvPathLength__L_PerCaS | 0.0008637308 | 0.5916009 | 0.538023 |
| NodeBWCent__L_MTG | 0.000428039 | 0.4000983 | 0.8445812 |
| AvPathLength__L_InfOcG_S | 0.0007079528 | 0.049996 | 1.9808431 |
| NodeBWCent__R_CS | 0.0005700522 | 0.732848 | 0.3421693 |
| AvPathLength__R_InfPrCS | 0.0006151096 | 0.4971359 | 0.6811658 |
| NodeBWCent__L_InfFGTrip | 0.0002641259 | 0.2790149 | 1.0876706 |
| AvPathLength__L_InfTS | 0.000463549 | 0.4668332 | 0.730068 |
| NodeBWCent__L_PrCun | 0.000578013 | 0.8516433 | 0.1874434 |
| NodeBWCent__R_SupCirInS | 0.000519519 | 0.2098923 | 1.2609129 |
| NodeBWCent__L_Tpo | 0.0006405219 | 0.2851643 | 1.0737911 |
| NodeBWCent__L_SupFS | 0.0003323756 | 0.1007301 | 1.6546121 |
| AvPathLength__L_SupPrCs | 0.0003795567 | 0.8959603 | 0.1310557 |
| NodeBWCent__L_SupOcG | 0.0006029441 | 0.1567469 | 1.4254113 |
| NodeBWCent__L_FMarG_S | 0.0004941294 | 0.2996283 | 1.0419329 |
| NodeBWCent__R_InfFGTrip | 0.0002071648 | 0.4691462 | 0.7262744 |
| NodeBWCent__L_PRCG | 0.0004837791 | 0.0058443 | 2.8088183 |
| NodeBWCent__L_SupTGLp | 0.0005710263 | 0.6895121 | 0.4005262 |
| AvPathLength__R_InfFGOpp | 0.0002917402 | 0.6090979 | 0.512767 |
| AvPathLength__R_PosVCgG | 0.0003902571 | 0.4358497 | 0.7819521 |
| AvPathLength__R_TrTs | 0.0003742823 | 0.2822812 | 1.0802726 |
| AvPathLength__L_ACirIns | 0.0005635503 | 0.253704 | 1.1471418 |
| NodeBWCent__R_SupTGLp | 0.0005643784 | 0.0692834 | 1.8337069 |
| AvPathLength__L_InfPrCS | 0.0003895812 | 0.6345674 | 0.4765773 |
| AvPathLength__R_OcPo | 0.0003351892 | 0.5872989 | -0.5442853 |
| NodeBWCent__R_IntPS_TrPS | 0.0001961395 | 0.912869 | -0.1096615 |
| AvPathLength__L_JS | 0.0004079759 | 0.5434692 | 0.6093882 |
| AvPathLength__L_SupTS | 0.0003687163 | 0.8840551 | 0.1461548 |
| NodeBWCent__R_SupFG | 0.0002812839 | 0.2651615 | 1.1197311 |
| AvPathLength__R_TrFPoG_S | 0.0001987824 | 0.6230364 | -0.4928811 |
| NodeBWCent__L_Hip | -1.68184e-05 | 0.4010689 | -0.8428374 |
| AvPathLength__R_ACirIns | 0.000366298 | 0.0878444 | 1.7215257 |
| AvPathLength__R_Cun | 0.000360596 | 0.9285999 | -0.0898031 |
| AvPathLength__R_ShoInG | 0.0002477075 | 0.1916472 | 1.313446 |
| NodeBWCent__L_HG | 0.0001771228 | 0.2125041 | 1.2536713 |
| NodeBWCent__L_Pu | 0.0001595881 | 0.5944141 | 0.5339394 |
| NodeBWCent__L_InfCirIns | 0.0002540914 | 0.3661217 | 0.9073451 |
| AvPathLength__L_Amg | -0.0001996052 | 0.8570054 | -0.1805924 |
| AvPathLength__L_FMarG_S | -0.0003568866 | 0.2131851 | -1.251794 |
| AvPathLength__R_PaCL_S | -0.0001411353 | 0.8741536 | -0.1587381 |
| AvPathLength__R_AOcS | -0.0004759372 | 0.0586744 | -1.9096126 |
| NodeBWCent__R_InfTS | 0.0001064558 | 0.5679843 | 0.5726727 |
| NodeBWCent__L_MOcG | -0.0004448476 | 0.1116088 | -1.6033124 |
| NodeBWCent__L_SupFG | -0.0002517965 | 0.5374476 | 0.6185325 |
| AvPathLength__L_TrFPoG_S | 0.000391063 | 0.8599587 | -0.1768227 |
| AvPathLength__L_CaN | -0.000211177 | 0.4384569 | -0.7775059 |
| AvPathLength__R_InfTS | -0.0001142687 | 0.2497247 | -1.1568656 |
| NodeBWCent__R_ATrCoS | -0.0001637605 | 0.0598592 | -1.9005899 |
| NodeBWCent__L_PaCL_S | -0.0002327247 | 0.5392911 | -0.6157276 |
| AvPathLength__L_InfFGOrp | -0.0002256553 | 0.69713 | 0.3901735 |
| NodeBWCent__R_Hip | -0.000388764 | 0.7521676 | 0.3165386 |
| NodeBWCent__L_PaHipG | -0.0004278795 | 0.5103618 | -0.6603312 |
| NodeBWCent__R_SbPS | -0.0002247261 | 0.7591009 | -0.3073921 |
| AvPathLength__L_InfFGTrip | -0.0004037002 | 0.2284636 | -1.2107763 |
| NodeBWCent__R_Tha | -0.0002894307 | 0.4789275 | -0.7103462 |
| AvPathLength__L_OcPo | -0.0005110448 | 0.3719004 | -0.8964214 |
| AvPathLength__R_PaHipG | -0.000446397 | 0.7399951 | -0.3326619 |
| AvPathLength__R_IntPS_TrPS | -0.0002244664 | 0.5652254 | -0.5767651 |
| AvPathLength__R_SupPrCs | -0.0003166583 | 0.3299016 | -0.9784583 |
| AvPathLength__R_PerCaS | -0.000481101 | 0.0093628 | -2.6430109 |
| NodeBWCent__R_MPosCgG_S | -0.0004339122 | 0.3756011 | -0.8894819 |
| NodeBWCent__R_OcPo | -0.0003720792 | 0.9782904 | -0.0272716 |
| NodeBWCent__L_CS | -0.0004747164 | 0.6791227 | -0.4147156 |
| AvPathLength__R_SbOrS | -0.0004652099 | 0.0774365 | -1.7817068 |
| NodeBWCent__R_SupFS | -0.0002436541 | 0.6366603 | -0.4736319 |
| NodeBWCent__L_ACgG_S | -0.0005194537 | 0.8344359 | -0.2094906 |
| NodeBWCent__R_MFS | -0.0002005376 | 0.6487003 | 0.4567667 |
| NodeBWCent__R_PosCG | -0.0005665221 | 0.6046272 | -0.5191882 |
| NodeBWCent__R_PosCS | -0.0005950305 | 0.0692979 | -1.8336098 |
| NodeBWCent__L_PosCG | -0.0003916269 | 0.1377684 | -1.4945595 |
| NodeBWCent__L_CaN | -0.0002424993 | 0.0463734 | -2.0137213 |
| AvPathLength__R_PosTrCoS | -0.000497801 | 0.1452779 | -1.4663656 |
| NodeBWCent__L_TrFPoG_S | -0.0003733082 | 0.5716848 | -0.5671986 |
| NodeBWCent__R_AngG | -0.0004960607 | 0.4646458 | -0.7336653 |
| AvPathLength__L_InfCirIns | -0.0005939149 | 0.0244062 | -2.2807316 |
| NodeBWCent__R_TrFPoG_S | -0.000514884 | 0.4761666 | -0.7148237 |
| AvPathLength__L_MACgG_S | -0.0003941872 | 0.0829239 | -1.7492025 |
| AvPathLength__R_InfFGOrp | -0.0006213989 | 0.4141841 | -0.8195216 |
| AvPathLength__R_PrCun | -0.0005282494 | 0.0411581 | -2.0651356 |
| NodeBWCent__R_MOcG | -0.0002556548 | 0.3078318 | -1.0243248 |
| NodeBWCent__L_RG | -0.0005457769 | 0.6067252 | -0.5161723 |
| AvPathLength__L_PosDCgG | -0.0007125311 | 0.1134375 | -1.5950865 |
| AvPathLength__R_LoInG_CInS | -0.0008965826 | 0.170343 | -1.3797417 |
| AvPathLength__L_MPosCgG_S | -0.0008702007 | 0.0139718 | -2.4961668 |
