# Supplementary material for: Machine learning model to predict obesity using gut metabolite and brain microstructure data
Source: Sci Rep. 2023 Apr 4;13:5488. doi: 10.1038/s41598-023-32713-2 (PMC10073225; doi:10.1038/s41598-023-32713-2)
Supplement: Supplementary file 2 — Supplementary Table 2. [file 41598_2023_32713_MOESM2_ESM.docx]

SuppTable2

| Column Name | SVM Weight | P-Value | T-Value |
| --- | --- | --- | --- |
| (R)-salsolinol | -0.062086442 | 0.2229156 | -1.225434 |
| 1,2-dilinoleoyl-GPC (18:2/18:2) | 0.1181861285 | 0.8407669 | 0.2013676 |
| 1-linolenoylglycerol (18:3) | 0.0588531672 | 0.5024215 | 0.6728043 |
| 1-oleoyl-GPC (18:1) | -0.1992198594 | 0.3254347 | -0.9875739 |
| 1-oleoylglycerol (18:1) | 0.0833233592 | 0.2049111 | 1.2749096 |
| 11beta-hydroxyandrosterone sulfate (2) | -0.1041779235 | 0.1132935 | -1.5957305 |
| 12-ketolithocholate | -0.1063555081 | 0.6234562 | -0.4922853 |
| 2'-deoxyadenosine 5'-monophosphate | 0.0198369654 | 0.785891 | -0.2722852 |
| 2-(4-hydroxyphenyl)propionate | 0.0222074854 | 0.2969804 | 1.0476857 |
| 2-hydroxypalmitate | -0.116926237 | 0.0279664 | -2.2259563 |
| 3,5-dihydroxybenzoic acid | -0.0216844786 | 0.2652425 | -1.1195405 |
| 3,7-dimethylurate | -0.0732102756 | 0.7599397 | -0.3062874 |
| 5-(2-Hydroxyethyl)-4-methylthiazole | 0.0298703752 | 0.9918073 | 0.0102906 |
| 5-hydroxyhexanoate | -0.0982964968 | 0.3068464 | -1.026423 |
| 5alpha-androstan-3alpha,17beta-diol disulfate | -0.0467398429 | 0.2266135 | -1.2156354 |
| 5alpha-androstan-3beta,17beta-diol monosulfate (1) | -0.04324831 | 0.3820199 | -0.8775459 |
| 7-methylxanthine | -0.0702359421 | 0.5315353 | -0.6275615 |
| acesulfame | 0.1599305826 | 0.222516 | 1.2264998 |
| AMP | 0.050813927 | 0.2627772 | -1.1253659 |
| agmatine | -0.0881268051 | 0.1105004 | -1.6083507 |
| arabitol/xylitol | -0.0843869419 | 0.1675273 | -1.3889653 |
| carboxyibuprofen | -0.0758462186 | 0.4496436 | -0.7586004 |
| curcumin | -0.0454703424 | 0.1972666 | -1.2968855 |
| deoxymugineic acid | -0.0815480139 | 0.2403965 | -1.1800981 |
| docosadienoate (22:2n6) | -0.0213110778 | 0.2522873 | -1.1505912 |
| docosahexaenoate (DHA; 22:6n3) | 0.0561603392 | 0.5487737 | 0.6013752 |
| docosahexaenoylcarnitine (C22:6)* | -0.0780151081 | 0.0613478 | -1.8894642 |
| gamma-glutamylalanine | 0.0551791078 | 0.1697141 | 1.3817916 |
| genistein | 0.0169121201 | 0.6114582 | 0.5093853 |
| glycocholate | -0.1375898656 | 0.2450839 | -1.1683452 |
| hyocholate | -0.0749421174 | 0.4151871 | -0.817757 |
| I-urobilinogen | 0.0373969979 | 0.0195845 | 2.36738 |
| imidazole propionate | -0.096304554 | 0.9937212 | -0.0078865 |
| levulinate (4-oxovalerate) | 0.0977410372 | 0.2314901 | 1.2028887 |
| linolenoyl-linolenoyl-glycerol (18:3/18:3) [2]* | -0.0272687861 | 0.3730772 | -0.89421 |
| linoleoyl-linoleoyl-glycerol (18:2/18:2) [1]* | 0.0907238206 | 0.0342553 | 2.1425781 |
| maleate | -0.0314907931 | 0.1094215 | -1.6132939 |
| maltose | 0.0515397106 | 0.3679204 | -0.9039334 |
| N-(2-furoyl)glycine | 0.0385985301 | 0.3071521 | 1.0257716 |
| N-acetylisoleucine | 0.119875234 | 0.02041 | 2.3512972 |
| N-butyryl-leucine | 0.0341935406 | 0.0931012 | 1.6933227 |
| N-methylproline | -0.0869607105 | 0.3443564 | -0.9495022 |
| N-stearoyl-sphinganine (d18:0/18:0)* | -0.1005999527 | 0.9127752 | 0.10978 |
| nicotinate ribonucleoside | -0.1216411215 | 0.5345713 | -0.6229187 |
| OAHSA (18:1/OH-18:0) | 0.0454157065 | 0.7071483 | 0.3766223 |
| pantoate | -0.100436776 | 0.0844496 | -1.7404801 |
| paraxanthine | -0.0431451772 | 0.2649834 | 1.1201508 |
| piperine | 0.0245568669 | 0.5037286 | 0.6707439 |
| pregnen-diol disulfate* | -0.1368044899 | 0.0766891 | -1.7862797 |
| stachydrine | -0.0799057873 | 0.9693121 | 0.0385549 |
| stigmastadienone | 0.0855641217 | 0.4839874 | 0.7021772 |
| sucralose | 0.1146917169 | 0.116412 | 1.5819302 |
| sulfate of piperine metabolite C16H19NO3 (2)* | 0.0535064384 | 0.1394363 | 1.488196 |
| theobromine | -0.0803158176 | 0.8264974 | -0.219696 |
| TMP | -0.0215154662 | 0.6429309 | -0.4648317 |
| tyrosol | 0.031158638 | 0.0755582 | 1.7932698 |
| Urolithin A | -0.0224049416 | 0.438942 | -0.7766804 |
